# Neuroendocrine Tumor of the Ampulla of Vater: A Case Report

**DOI:** 10.7759/cureus.38588

**Published:** 2023-05-05

**Authors:** Mohammad S Aman, Bidhan C Das, Aminul Islam, Murshidul Arefin, Satya N Gupta

**Affiliations:** 1 Department of Hepatobiliary, Pancreatic and Liver Transplant Surgery, Bangabandhu Sheikh Mujib Medical University (BSMMU), Dhaka, BGD

**Keywords:** bangladesh, duodenal malignancy, whipple procedure, immunohistochemistry, pancreatico-duodenectomy, double-duct sign, ampulla of vater, neuroendocrine tumor

## Abstract

Neuroendocrine tumors (NETs) of the ampulla of Vater are extremely rare. Here, we discuss the clinical presentation, diagnostic challenges, and treatment options of a recently experienced case of NET of the ampulla of Vater in light of the literature. A 56-year-old woman presented with recurrent upper abdominal pain. Ultrasonography (USG) of the whole abdomen showed multiple gallstones along with a dilated common bile duct (CBD). For evaluating the dilated CBD, a magnetic resonance cholangiopancreatography was performed, which revealed the double-duct sign. Subsequently, an upper gastrointestinal endoscopy showed a bulged-out ampulla of the Vater. Biopsy and histopathological examination of the growth yielded the diagnosis of adenocarcinoma. A Whipple procedure was performed. Macroscopically, a 2 cm growth was noted involving the ampulla of Vater, and microscopic findings were consistent with a well-differentiated NET, grade 1 (low grade). The diagnosis was further confirmed by immunohistochemical staining (pan-cytokeratin positive, synaptophysin positive, and focally chromogranin positive). Her postoperative course was uneventful except for delayed gastric emptying. A detailed evaluation and a high index of suspicion are required for the diagnosis of this rare tumor. Treatment is relatively easier after a proper diagnosis.

## Introduction

Neuroendocrine neoplasms (NENs) arise from cells of the diffuse neuroendocrine system [1,2]. These are typically located in the gastrointestinal (GI) tract, bronchopulmonary tract, thymus, and pancreas [1]. According to the 2019 World Health Organization (WHO) classification (fifth edition), NENs are divided into two types, namely, well-differentiated neuroendocrine tumors (NETs) and poorly differentiated neuroendocrine carcinomas (NECs). NETs are further divided into the following three grades based on mitotic rate and Ki-67 proliferation index: low-grade (G1), intermediate-grade (G2), and high-grade (G3) [1,3]. A higher mitotic rate and a higher Ki-67 index are associated with a more aggressive clinical course and a poor prognosis. These parameters, along with tumor differentiation, are important when considering the treatment protocol of patients with gastro-entero-pancreatic NET (GEP-NET).

NETs are essentially rare diseases, and therefore, data related to their epidemiology are limited. Despite the fact that GEP-NETs account for two-thirds of all NETs, the duodenum is a rare location for this disease [4,5]. According to a recent Japanese study of 33,215 NEN patients, just about 9% of NETs arise in the duodenum [6]. Among these, NETs of the ampulla of Vater are extremely rare. Currently, there is no clear data revealing the exact percentage of ampullary NENs/NETs. These rarities make it challenging to predict an ampullary NET preoperatively. In this context, surgeons have to face certain challenges if unexpected findings are discovered in a resected specimen after surgery [7].

Here, we report a recently encountered case of a 56-year-old woman with cholelithiasis and double-duct sign, i.e., simultaneous dilatation of the common bile duct (CBD) and the main pancreatic duct (MPD). The Vater was examined and revealed a growth. The biopsy was suggestive of adenocarcinoma. However, after the Whipple procedure, both histopathology and immunohistochemistry were confirmatory of a well-differentiated NET.

## Case presentation

A 56-year-old diabetic, hypertensive, post-menopausal woman presented with repeated episodes of right upper abdominal pain for the last one and a half years. The pain was associated with anorexia, nausea, and occasional non-bilious vomiting. She also reported flatulence and a sensation of fullness after meals for the same duration. Initially, she visited her family physician, who performed an abdominal ultrasonography scan and found multiple gallstones along with a dilated CBD (12.7 mm). Cholecystectomy was advised, and she was referred to our department.

At presentation, besides the above-mentioned complaints, she gave no history of significant weight loss, fever, jaundice, diarrhea, hematemesis, melena, dyspnea, chest pain, or bone pain. Her bowel and bladder habits were regular. She was mildly anemic but not icteric. Her vitals were stable. The abdomen was soft and non-tender. No masses or hepatosplenomegaly were detected. Her bowel sounds were normal. The remainder of the examination was also normal. Her hemoglobin level, leukocyte count, liver functions, and renal functions were within normal limits. All her tumor and viral markers were also found to be normal.

**Figure 1:**
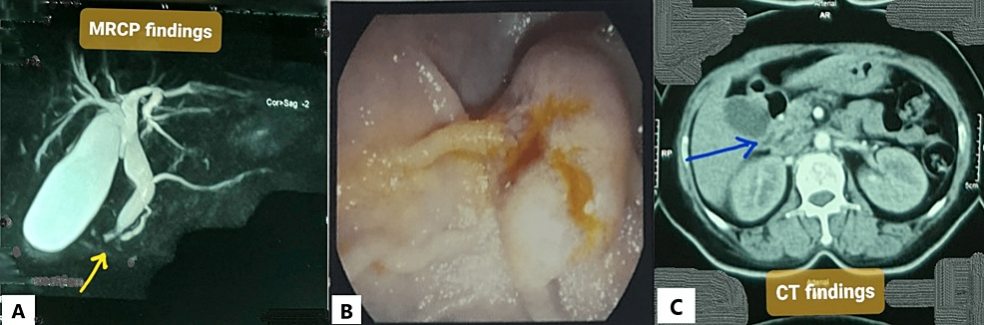
(A) MRCP findings suggestive of dilated CBD and MPD also known as the double-duct sign (yellow arrow). (B) UGI endoscopy showing the bulged-out ampulla. (C) Axial view of the CECT of the chest and upper abdomen showing an ill-defined, minimally enhancing soft tissue lesion in the periampullary area with dilated MPD (blue arrow). MRCP: magnetic resonance cholangiopancreatography; CBD: common bile duct; MPD: main pancreatic duct; UGI: upper gastrointestinal; CECT: contrast-enhanced computed tomography

A magnetic resonance cholangiopancreatography (MRCP) was done, and it showed a distended gallbladder with calculi and sludge, as well as dilated CBD and MPD (double-duct sign) (Figure 1A). A UGI endoscopy was done to check the ampullary region. During the procedure, the ampulla was found to be swollen and ulcerated (Figure 1B). A forceps biopsy was obtained from the ampulla. Histopathology revealed that lamina propria in some areas contained signet ring cells, and some glands were lined by atypical epithelium, indicating adenocarcinoma, grade II. A review of the histopathology revealed similar malignant findings. To stage the disease, contrast-enhanced computed tomography (CECT) of the chest and abdomen was performed. It revealed an ill-defined, minimally enhancing soft tissue lesion in the periampullary area, similar to prior investigations (Figure 1C). The lesion caused biliary tree dilatation as well as mild pancreatic duct dilatation. The positron emission tomography scan was postponed due to the low socioeconomic condition of the patient.

**Figure 2:**
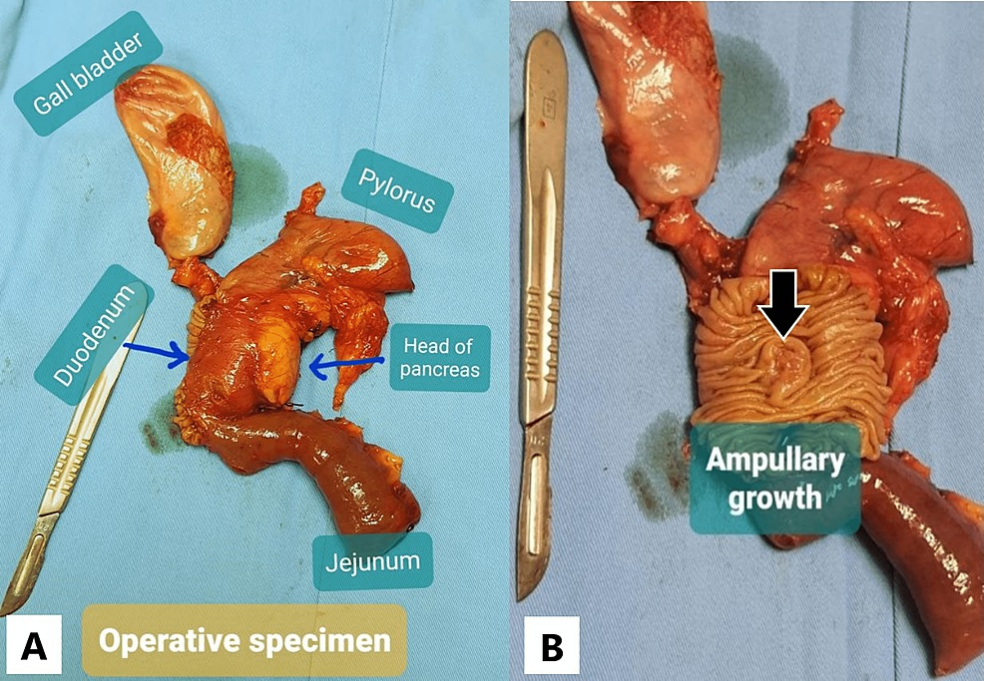
(A) Operative specimen (all major parts are marked separately within the image). (B) The ampullary growth (black arrow). *: The scalpel was kept on the side for measurement.

Considering the history and investigation data, the patient was adequately counseled, and Whipple’s pancreaticoduodenectomy was performed (Figure 2A). Macroscopically, there was a periampullary growth in the second part of the duodenum with a maximum diameter of 2 cm (Figure 2B).

**Figure 3:**
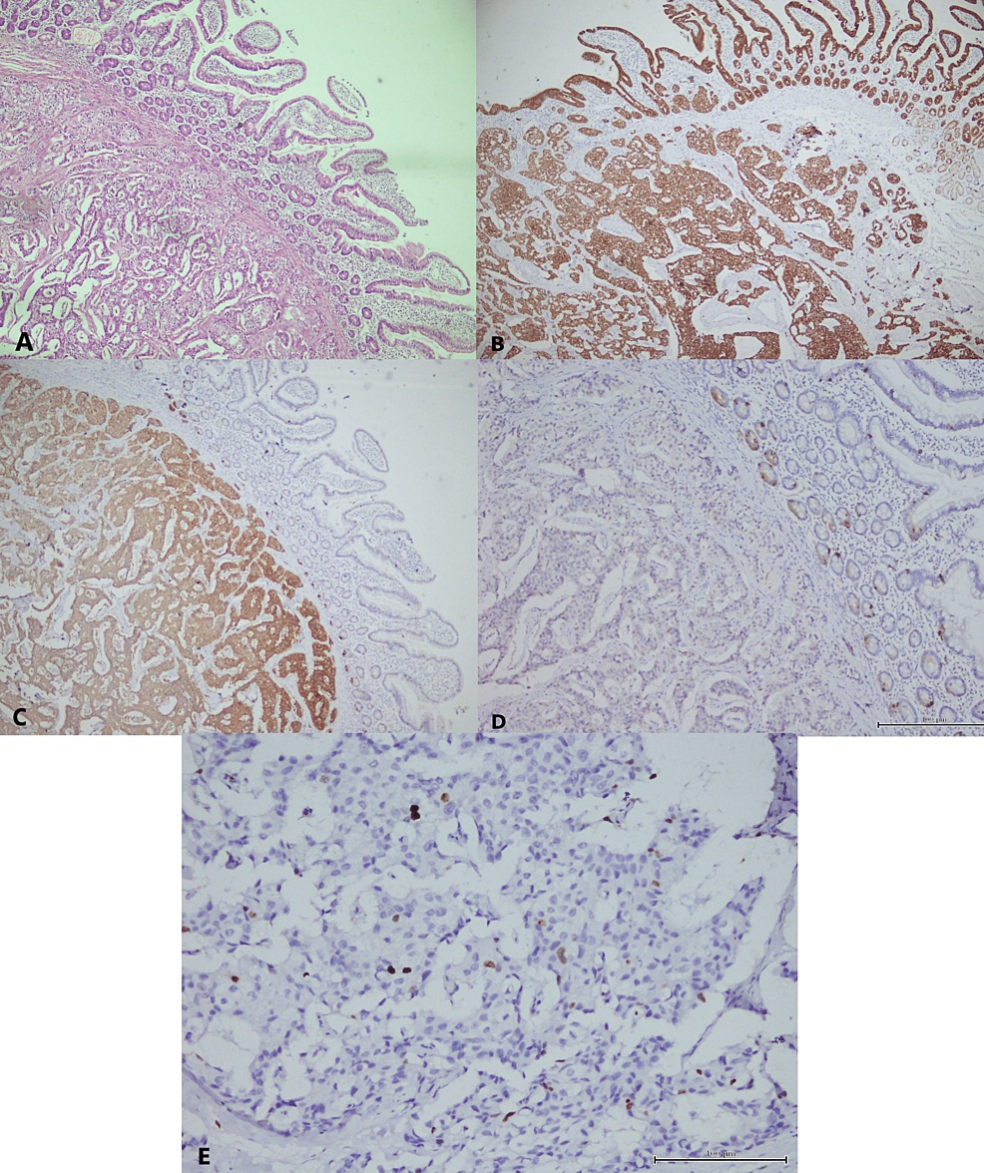
(A) Hematoxylin and eosin stain of the ampullary lesion with overlying intact duodenal mucosa. Cells of the lesion are arranged in nests, glands, trabeculae, and cords. (B) Pan-cytokeratin positivity of the lesion and overlying mucosa. (C) Diffuse positivity of synaptophysin of the lesion. (D) Focal positivity of chromogranin A of the lesion. (E) Fewer than 2% of cells show positivity for Ki-67.

Microscopically, the periampullary region was lined by duodenal mucosa. The submucosa showed a neoplasm composed of cells arranged in nests, glands, trabeculae, and cords. It was infiltrating into the muscularis propria (Figure 3A). All resected margins were free. There were no lymphovascular or perineural invasions. The tumor was unifocal with a mitotic count of <2 mitoses/2 mm^2^. Five lymph nodes were dissected, of which one showed metastasis. On immunohistochemistry studies, the tumor cells were positive for pan-cytokeratin (Figure 3B), strongly and diffusely positive for synaptophysin (Figure 3C), and focally positive for chromogranin (Figure 3D). Only 2% of tumor cells were positive for Ki-67 (Figure 3E). Based on the histopathological and immunohistochemistry findings, the diagnosis of a well-differentiated NET, G1 (low grade), was established. According to the American Joint Committee on Cancer eighth edition, it was pT2N1M0.

The patient recovered well from anesthesia. She developed hematemesis once on the first postoperative day (POD) and was monitored intensively. As her condition improved, her oral diet was started on the fourth POD. However, she developed delayed gastric emptying with persistent vomiting. The nasogastric tube was reinserted on the seventh POD for gastric decompression and kept for five days. Her oral diet was restarted on the 12th POD, and this time she tolerated her feeding well. All drain tubes were removed by then. She was discharged on the 17th POD with advice for adjuvant chemotherapy.

## Discussion

Duodenal NENs (d-NENs) are mostly (>90%) found in the first and second parts of the duodenum. Overall, 20-40% of these occur around the periampullary region [8-12]. Vanoli et al. conducted a study on 203 d-NENs cases and found 77 (39%) of them occurred in the ampullary area. Among them, 55 were NETs, and 22 were NECs [11]. Chen et al. found that among all d-NENs, there were significant differences in characteristics between ampullary NENs and non-ampullary d-NENs. The median size of the ampullary NENs is 2.5 cm compared to 1.6 cm for non-ampullary d-NENs in the Asian cohort. Thus, ampullary lesions are larger, more frequently present with jaundice, more commonly detected as poorly differentiated NECs, and more likely to have lymphatic and distant metastases [12].

The mean age of presentation of d-NENs is in the sixth decade, with a slight male predominance [4,6,9]. The majority (90%) of d-NENs are not associated with a functional syndrome. In the case of non-ampullary d-NENs, patients most commonly present with pain, bloating, anemia, and, less commonly, nausea, vomiting, diarrhea, and duodenal obstruction. However, in the case of ampullary NENs, patients usually present with obstructive jaundice, pain, bile duct dilation, vomiting, and diarrhea [9]. Our patient presented with abdominal pain, nausea, vomiting, and a double-duct sign.

According to Berge et al., the double-duct sign is a strong predictor of the presence of malignancy, but its clinical utility is limited due to its poor negative predictive value (47%) and intermediate sensitivity (72%) and specificity (73%) [13]. However, Krishna et al. [14] and Sinha et al. [15] have shown that the rate of malignancy is significantly lower in patients who do not have obstructive jaundice, yet it is clinically important and warrants additional diagnostic assessment. It must be evaluated with UGI endoscopy and biopsy in individuals with periampullary lesions to distinguish premalignant lesions from invasive carcinoma. Unfortunately, endoscopic biopsies are also, by definition, superficial, and, therefore, the risk of sampling error exists. As demonstrated by Berge et al., these also have both low sensitivity (71%) and low negative predictive value (51%) [13]. Regardless of the results of biopsies, physicians must be aware of the increased possibility of a malignant cause in the presence of a double-duct sign. However, histopathology of the resected specimen is always necessary to establish the final diagnosis [7]. For all NENs, immunohistochemistry for synaptophysin and chromogranin-A is the bare minimum of tests to support the diagnosis. In these circumstances, the mitotic count and the Ki-67 proliferation index should be noted.

Our patient did not have jaundice. Yet, before embarking on surgery for cholelithiasis, due to strong suspicion of ampullary malignancy evidenced by the double-duct sign on MRCP, we performed a UGI endoscopy with biopsy and subsequent histopathology. As NENs have glandular components such as adenomas and adenocarcinomas, our forceps biopsy during UGI endoscopy may have failed to obtain the complete tissue architecture needed for histopathology. It created a diagnostic dilemma. However, after the Whipple procedure, as soon as we received the histopathology report establishing the diagnosis of a well-differentiated NET, we asked for immunohistochemistry from a well-reputed center for further clarification. All markers (pan-cytokeratin, synaptophysin, and chromogranin-A) were positive in our case, supporting the histopathology. Besides, the mitotic count of <2 mitoses/2 mm2 and the Ki-67% index of <3% jointly confirmed the G1 (low grade) nature of the lesion as per the new WHO classification (2019) [3].

The recommended treatment for d-NENs is resection (level of evidence 4). Endoscopic resection is the treatment of choice for well-differentiated, localized, non-metastatic tumors less than 1 cm in size and confined to the submucosa layer. Whereas surgery is preferred for lesions extending beyond the muscular layer, lesions larger than 1 cm in size, and all cases of lymph node metastases (level of evidence 5) [10]. The best possible surgery is a pancreaticoduodenectomy. As in our case, the lesion was 2 cm in size, invaded the muscle layer (T2), and had lymph node metastasis. The Whipple procedure was ideal for our patient, and we also recommend it for similar cases in the future.

Vanoli et al. suggested that in the large population of non-functioning NETs, only those ≥2 cm in size appeared to be at constant risk of both local lymph node and distant metastases, while those <1 cm appeared to carry a negligible risk [11]. However, from John Hopkins Hospital, Dogeas et al. reported a slightly different opinion. Among 101 d-NETs patients, 56 had lymph node evaluation, and 27 (48%) of those had positive lymph nodes. To be more precise, the lymph node positivity (LN+) rate for tumors with a diameter of <1 cm was 4.5% (1/22), 72% (13/18) for tumors between 1 and 2 cm, and 81% (13/16) for tumors >2 cm. Only tumor size, not tumor grade or depth of invasion, predicted the likelihood of LN+ in both univariate and multivariate analyses. They concluded that duodenum and periampullary NETs frequently involve the lymph nodes, especially those that are larger than 1 cm. These larger tumors (>1 cm) were recommended to be resected with lymphadenectomy [8]. This study justifies the Whipple procedure for our patient as she had a 2 cm lesion and there was lymph node involvement (one out of five) in the resected specimen.

According to Dogeas et al., the overall survival (OS) rates among d-NETs patients were 89% at one year, 87% at three years, and 79% at five years. The median survival duration was 114 months. However, no differences in OS were observed between the primary tumor site or the extent of resection [8].

## Conclusions

NET in the ampullary region is an extremely rare tumor and its diagnosis is challenging. Systemic evaluation and a very high index of suspicion are required for diagnosis. Treatment decision is relatively easier once the diagnosis is established.
